# Giant adrenal endothelial cyst associated with acute and chronic morbidity in a young female: a case report

**DOI:** 10.4076/1757-1626-2-8841

**Published:** 2009-09-09

**Authors:** Muhammad Rizwan Khan, Saad Ajmal, Taimur Saleem

**Affiliations:** 1 Department of Surgery, Aga Khan University, (Stadium Road), P.O. Box 3500, Karachi, (74800), Pakistan; 2 Medical College, Aga Khan University, (Stadium Road), P.O. Box 3500, Karachi, (74800), Pakistan

## Abstract

Adrenal cysts are rare clinical entities that can present as acute abdomen through rupture and internal hemorrhage as well as chronic symptoms such as gastrointestinal disturbances. A 20-year-old girl presented to our hospital with a 4-years history of abdominal pain and diarrhea. Ultrasound of the abdomen revealed a cystic area measuring 10 × 10 cm between the spleen and left kidney. Computed tomography scan showed a large cystic, homogeneous mass measuring 12.8 × 9.5 × 9.4 cm in the left hypochondrium with most likely origin from the left adrenal gland. Limited work up for hormone hypersecretion was negative. The patient was then encountered in the emergency room with an acute abdomen secondary to intracystic hemorrhage. A laparotomy with left adrenalectomy was performed. Final pathology showed a benign adrenal endothelial cyst. Post-operatively, the patient's long standing complaints of diarrhea and abdominal pain completely resolved. Surgical resection appears a safe and reasonable management strategy in a patient with intracystic hemorrhage of adrenal cyst.

## Introduction

Adrenal cysts are rarely encountered clinical entities. Their incidence has been reported from 0.06%-0.18% in autopsy series [1]. Although most of these cysts are asymptomatic, they can cause morbidity in patients in the acute as well as chronic settings through rupture and internal hemorrhage or gastrointestinal disturbances through pressure on various parts of the gastrointestinal tract, respectively [2]. However, the optimal management of adrenal cysts remains controversial.

We report here the case of a 20-years-old girl who presented to our institution with acute and chronic morbidity resulting from a giant non-functioning left adrenal cyst. She was managed with surgical intervention. We have also reviewed the indications for surgical intervention in a patient with an adrenal cyst.

## Case presentation

A 20-year-old Pakistani girl from Karachi presented to our hospital with a 4-years history of abdominal pain and diarrhea. She had been having up to 10 episodes of loose stools per day associated with abdominal heaviness and tenesmus. In view of her chronic symptoms, she had been empirically prescribed anti-tuberculous therapy in the past due to suspicion of abdominal tuberculosis. Her physical examination was unremarkable.

Laboratory investigations showed an increased erythrocyte sedimentation rate (37 mm/hr, normal: <20 mm/hr) while lipase, amylase, liver function tests, electrolytes and a stool detailed report (DR) were all within normal range. In view of her normal laboratory parameters, she next underwent an upper gastrointestinal endoscopy and a colonoscopy to further evaluate her chronic diarrhea. Patchy erythema in the ascending and transverse colon along with mild antral erythema in the stomach were noted. Biopsy of different parts of the digestive tract revealed chronic non-specific inflammation in the duodenum, ileum, ascending and descending colon with mild chronic *Helicobacter pylori* associated gastritis. Ultrasound of the abdomen revealed a cystic area measuring 10 cm × 10 cm between the spleen and left kidney.

**Figure 1:**
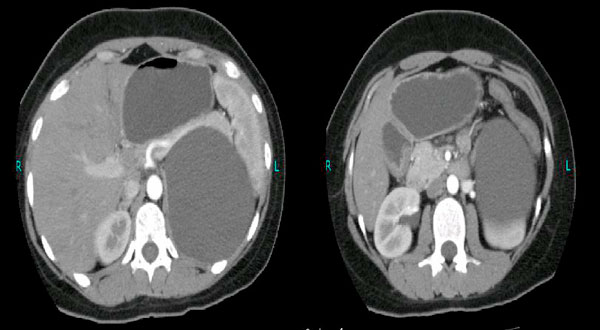
**Giant left adrenal cyst without any rupture or intracystic hemorrhage**.

Computed tomography (CT) scan showed a large cystic, homogeneous mass in the left hypochondrium with most likely origin from the left adrenal gland (Figure 1). The mass measured 12.8 × 9.5 × 9.4 cm in cranio-caudal, antero-posterior and transverse dimensions and was abutting various parts of the gastrointestinal tract. Urinary vanillyl mandelic acid (VMA) and 5-hydroxyindoleacetic acid (5-HIAA) were done to rule out endocrine tumors of the adrenal gland. These were, however, within normal limits.

**Figure 2:**
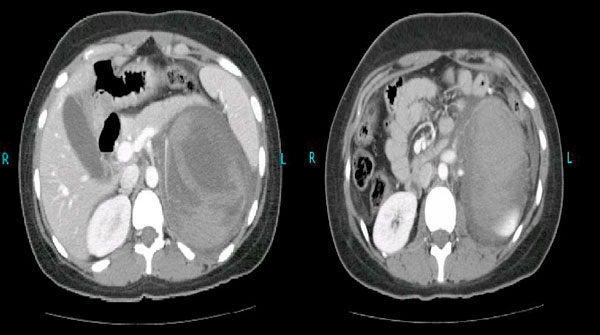
**Giant left adrenal cyst complicated by intracystic hemorrhage**.

While the work up was in progress, the patient was encountered in the emergency room of the hospital with one day history of excruciating abdominal pain. On examination, she had an acute abdomen which was exquisitely tender in the left upper quadrant with a vaguely palpable mass in the umbilical area. Surgical intervention was planned in view of the quickly decreasing hematocrit. Multiple blood transfusions were given and a CT scan was emergently performed which revealed a left adrenal gland cyst with internal hemorrhage (Figure 2). Moderate amount of free fluid was also present in the peritoneal cavity around this cyst.

After stabilization, the patient was taken to the operation theatre where a laparotomy with left adrenalectomy was undertaken. Histopathological examination of the specimen showed cystic tissue with no definite lining and fibro-collagenous wall exhibiting areas of hemorrhage and congestion. Immunohistochemical staining for CD31 was positive; confirming the diagnosis of a benign adrenal endothelial cyst.

The post-operative hospital course of the patient was unremarkable and she was discharged after a week. She has been maintaining regular follow-ups in the clinic and her long standing complaints of diarrhea and abdominal pain have completely resolved.

## Discussion

Adrenal cysts are generally rare and around 600 cases of adrenal cysts have been reported so far [3,4]. Approximately 7% of all adrenal cysts are malignant or potentially malignant [4].

Adrenal cysts may be categorized as any of the four major types including pseudocysts (39%), epithelial (9%), parasitic (7%; generally echinococcal) or endothelial cysts (45%) [2,5] Endothelial cysts are further classified into angiomatous and lymphangiomatous cysts depending in their immunohistochemical staining pattern. [6]

Adrenal cysts larger than 10 cm are rare [2,7]. Giant adrenal cysts generally pose a diagnostic conundrum for the surgeon because of the difficulty in localizing the origin of the cyst. Close proximity of the cyst to several important structures confounds the diagnosis. Literature review revealed a case report of a giant adrenal cyst which had initially been mistaken for a pancreatic pseudocyst and the correct diagnosis was made intra-operatively [8]. Our patient had a giant left adrenal cyst which was first noted on ultrasound. CT scan was performed next which helped in better localization of the cyst and delineated its possible origin.

The management of the patient with an adrenal cyst includes the battery of history taking, physical examination complemented by appropriate laboratory investigations to rule out a functioning lesion. CT scan and aspiration of the cyst may be performed for better characterization of the lesion [4].

Most of the adrenal cysts are asymptomatic and encountered as "incidentalomas" [2]. Adrenal cysts have been reported to account for 6% of all adrenal incidentalomas [9]. Expectant observation appears a reasonable approach for such asymptomatic lesions, especially if the size is small.

Intervention in the form of excision or aspiration for an adrenal cyst is indicated if the cysts are symptomatic, rapidly expanding, producing endocrine abnormalities, hemorrhagic, when malignancy can not be ruled out unequivocally or if their size exceeds 5 cm [2,10]. Our patient had chronic symptoms such as diarrhea and abdominal pain most likely resulting from pressure of the giant adrenal cyst on the gastrointestinal tract. Surgical intervention was mandated when she presented acutely with severe abdominal pain secondary to internal hemorrhage and presumed rupture of the adrenal cyst. Intracystic hemorrhage has been described as a rare but life-threatening complication of adrenal cyst that may lead to hypovolemic shock [11]. The operation in such circumstances is usually an open adrenalectomy because of the difficulty in controlling active bleeding inside large masses with the laparoscopic approach [12].

## Conclusion

In summary, follow-up of patients with asymptomatic adrenal cysts is needed to document the incidence of conversion of the cyst to a symptomatic form and occurrence of hemorrhage in such cysts. The documentation of such risks will help in providing an evidence based justification for surgical intervention in the management of asymptomatic adrenal cysts. Acute complications arising in benign adrenal cysts can be safely managed by surgical excision.

## Consent

Written, informed consent was obtained from the patient for the publication of this case report and accompanying images. A copy of the consent form is available for review by the Editor-in-Chief of the journal.

## Competing interests

The authors declare that they have no competing interests.

## Authors' contributions

SA and TS collected the data, helped in its interpretation and drafted the manuscript. MRK conceived the study, helped in the interpretation of the data, drafted the manuscript and provided overall supervision in the project.
